# Are Religiosity and Spirituality Related to Self-Reported Health Expectancy? An Analysis of the European Values Survey

**DOI:** 10.1007/s10943-021-01348-w

**Published:** 2021-07-20

**Authors:** Gillian Libby, Zachary Zimmer, Andrew Kingston, Clove Haviva, Chi-Tsun Chiu, Mary Beth Ofstedal, Yasuhiko Saito, Carol Jagger

**Affiliations:** 1 Leeds Institute of Rheumatic and Musculoskeletal Medicine, Leeds University, Leeds, UK; 2 Global Aging and Community Initiative, Mount Saint Vincent University, Halifax, Canada; 3 Population Health Sciences Institute, Newcastle University, Campus for Ageing and Vitality, Newcastle upon Tyne, NE4 5PL UK; 4 Geriatric Medicine Research, Department of Medicine, Dalhousie University, Halifax, Canada; 5 Institute of European and American Studies, Academia Sinica, Taipei, Taiwan; 6 Institute for Social Research, University of Michigan, Ann Arbor, USA; 7 College of Economics and Population Research Institute, Nihon University, Tokyo, Japan

**Keywords:** Health expectancy, Religion, Spirituality, Self-rated health, Comparative research

## Abstract

Research on religiosity and health has generally focussed on the United States, and outcomes of health or mortality but not both. Using the European Values Survey 2008, we examined cross-sectional associations between four dimensions of religiosity/spirituality: attendance, private prayer, importance of religion, belief in God; and healthy life expectancy (HLE) based on self-reported health across 47 European countries (*n* = 65,303 individuals). Greater levels of private prayer, importance of religion and belief in God, at a country level, were associated with lower HLE at age 20, after adjustment for confounders, but only in women. The findings may explain HLE inequalities between European countries.

**Supplementary Information:**

The online version contains supplementary material available at 10.1007/s10943-021-01348-w.

## Introduction

Research that links individuals’ religious and spiritual behaviours and beliefs with their health goes back a century (Dearmer, 1909; George et al., 2000; Hiltner, 1943; Koenig et al., 2012). The balance of this research shows that religion and spirituality relate to better health at all ages, but the associations in old age are particularly strong (Corsentino et al., 2009; Lawler-Row & Elliott, 2009). Frequency of attendance, and prayer, are two of the most common dimensions of religiosity and spirituality examined alongside health and mortality (Koenig, 2001; Powell et al., 2003). Frequency of attendance at religious services has the strongest empirical support for its association with physical health (George et al., 2002) and self-rated health (Nicholson et al., 2010). Private prayer is often used in conjunction with attendance in indices of religiosity, in spite of the two measures often having opposite relationships with health (Levin, 2013), with prayer’s negative correlation with health thought due to its use as a coping mechanism during illness (Masters & Spielmans, 2007). This association is particularly strong when prayer is undertaken outside of other religious activities (Ahrenfeldt et al., 2017). Importance of religion in one’s life has been used in multi-country comparisons of mortality (Stavrova, 2015), whilst belief in God, though central to religion, is infrequently used as a predictor of health (Exline, 2002). In a recent meta-analysis of the effect of religiosity and spirituality on physiological health, most measures of religiosity (including attendance and prayer) were positively associated with good health markers; however, intrinsic religiosity appeared to be negatively associated (Shattuck & Muehlenbein, 2020).

Nevertheless, such research has almost universally considered mortality outcomes separately from health outcomes, despite a longer life not necessarily meaning that more years are spent in good health. Health expectancy, the remaining number of years lived in a healthy state, is a population indicator that combines information on health and mortality. With population ageing, many countries have begun to move the focus from extending life expectancy to maximising health expectancy, more specifically disability-free life expectancy or healthy life expectancy (HLE) based on self-reported health, though obtaining comparative health data across countries has been problematic. The European Union has been a leader in this respect as, since 2005, it has monitored the health of its constituent countries by the Healthy Life Years (HLY) indicator, a disability-free life expectancy indicator based on the Global Activity Limitation Indicator (GALI) (Van Oyen et al., 2006).

The absence of research that examines religion and spirituality on one hand and the combination of morbidity and mortality, as in HLE, on the other represents a gap in the literature, and there are a number of reasons for assessing how religiosity and spirituality relate to HLE in Europe. Much of the evidence on religion and health has been based on data from the United States (Koenig et al., 2012; McCullough et al., 2000). Current European research provides some evidence of an association between religiosity and health, but cross-country comparisons are rare (Ahrenfeldt et al., 2017; Braam et al., 2001; Hank & Schaan, 2008; Nicholson et al., 2009). This paper is the first to focus on four dimensions of religiosity and spirituality: frequency of attendance at religious services, frequency of private prayer, importance of religion, and belief in God, and their relationship with HLE cross-sectionally across multiple European countries.

## Methods

Data for this study came from individuals aged 20 years and over taking part in the European Values Study (EVS), a large, representative, cross-national, and longitudinal survey research programme providing insights into what Europeans think about life, family, work, religion, politics, and society (EVS, 2008). We used the most recent data, the fourth wave (EVS, 2008), which comprises interviews of almost 70,000 individuals across 47 European countries.

### Measures

We selected questions from the EVS 2008 to tap four dimensions of religiosity: frequency of attendance at religious services, frequency of private prayer, importance of religion, and belief in God. Frequency of attendance was based on one question “Apart from weddings, funerals and christenings, about how often do you attend religious services these days?” with responses from “never, practically never” (0) to “more than once a week” (6). Private prayer was similarly measured by one question “How often do you pray to God outside of religious services?” with seven responses, from “never” (0) through “every day” (6). We assessed importance of religion by the sum of five questions: “How important is religion in your life?” with responses from “not at all important” (0) to “very important” (3), “Do you personally think it is important to hold a religious service for any of the following events? Birth, Marriage, Death” with “Yes/No” (1/0) responses for each, and “How important is God in your life?” with responses from “not at all important” (0) to “very important” (9). Belief in God was measured as the sum of two questions: “Do you believe in God? Yes/No” (1/0); and “Which of these statements comes closest to your beliefs?” with responses: “There is a personal God” (3), “There is some sort of spirit or life force” (2), “I don’t really know what to think” (1), and “I don’t really think there is any sort of spirit, God or life force” (0). Thus the range of scores for each dimension were: frequency of attendance (0–4); private prayer (0–4); importance of religion (0–15); belief in God (0–4), with higher scores denoting greater engagement. Full details of the questions and responses are provided in Supplementary Table 1.

The EVS contains only one survey item on health, the self-reported health question: “Describe your state of health these days; very good, good, fair, poor or very poor?” For calculation of healthy life expectancy, we defined good health as a response of good or very good, with the remainder (responses fair, poor or very poor) defined as not good.

Our analysis of healthy life expectancy adjusted for several measures at individual and country level that are important determinants of health, mortality, and religiosity (Cambois et al., 2016; Fouweather et al., 2015; Jagger et al., 2008; Nicholson et al., 2009). In individual-level analyses, we adjusted for highest level of education, categorised as lower (elementary or incomplete secondary), middle (completed secondary) or upper (tertiary), as a measure of socio-economic status. We chose to not adjust for denomination as our aim is to examine associations pertinent to broader societal belief systems rather than the impact of religious practices specific to separate denominations. As in other studies of multiple European countries (Nicholson et al., 2009), for country-level analyses we adjusted for three factors: the Gini index of inequality (scored 0 to 100 with higher scores indicating greater inequality), obtained from the World Development Indicators (2008); the proportion of individuals with a lower level of education, obtained from the EVS; and a measure of religious diversity based on the religious denomination question in the EVS, because, in general, religion has a positive effect on health in countries where there is religious freedom and diversity, and individuals are free to choose what and how they practice (44). Therefore, we calculated religious diversity by the Simpson Index of ecological diversity (Simpson, 1949), which has scores that can theoretically range from 0 indicating no diversity (all individuals having the same religious denomination) to 1 indicating maximum diversity (all individuals have a different denomination); “No affiliation” counted as a separate denomination.

### Statistical Methods

First, we explored whether the cross-sectional association between self-reported health and the dimensions of religiosity differed by country, by fitting ordinal logistic regression models adjusted for age, gender, and education, separately for each dimension and each country. For the ordinal regression models, self-reported health was grouped into three categories: good or very good, fair, poor, or very poor. We then used the Sullivan method (Sullivan, 1971) to calculate healthy life expectancy for each of the countries by applying the age- and sex-specific prevalence of fair, poor, or very poor health for that country to the relevant life tables calculated from population and mortality data from the Human Mortality Database (HMD, 2017). Finally, we used meta regression (Higgins & Green, 2008) to assess the contribution of religiosity and spirituality to healthy life expectancy across countries, summarising individual responses to each religious dimension with the mean for each country. The *p* values of the meta-regression models were adjusted for multiplicity by permutation tests (Higgins & Thompson, 2004). Three models were fitted for each religious dimension: Model 1, unadjusted; Model 2, adjusted for the Gini index of inequality and education; and Model 3, further adjusted for religious diversity. Healthy life expectancy could not be calculated for some countries due to unavailability of life tables (Kosovo, Northern Cyprus), and the Gini index of inequality was unavailable for others (Bosnia, Malta, Great Britain and Northern Ireland); therefore, meta-regression models were based on 41 of the 47 countries in the EVS. Standard regression models were used to assess the association between the dimensions of religiosity and life expectancy at age 20 (LE20). Analyses were performed separately for men and women. All analyses were undertaken in STATA 13.1.

### Sensitivity Analyses

We undertook a sensitivity analysis first for response rate as, with a small number of exceptions, the net sample size for each country was around 1500, but the total number of sample units issued varied. The meta-regression modelling was repeated therefore adjusting for EVS country-level response rate. Since the response rate for Germany was unavailable, we refitted Model 3 excluding Germany and then added response rates.

## Results

The EVS 2008 contained information on 65,319 individuals aged 20 to 108 years in the 47 countries. We excluded 16 individuals who were missing all nine variables used to compile the four dimensions of religiosity and spirituality. The remainder formed the analytic sample (*n* = 65,303) of whom 56% were women and the main religious denomination was Roman Catholic (Table 1). Religious diversity, as measured by Simpson’s Index, varied from 0.03 (Turkey) to 0.76 (Latvia). Missing data on self-reported health or education was below 1%.

**Table 1:** Socio-demographic, religiosity, and health characteristics of analytic sample

	(*n* = 65,303)
Sex % (*n*)	
Female	55.6 (36,311)
Male	44.4 (28,989)
Age % (*n*)	
< 50 years	55.8 (36,419)
≥ 50 years	44.2 (28,884)
Education level % (*n*)	
Lower (elementary or incomplete secondary)	29.3 (19,114)
Middle (complete secondary)	45.8 (29,947)
Upper (tertiary)	24.0 (15,682)
Not known	0.9 (560)
Religious denomination % (*n*)	
Roman catholic	27.8 (18,163)
Orthodox	23.1 (15,111)
Protestant	11.1 (7255)
Free church/Non-conformist/Evangelical	0.4 (278)
Muslim	11.3 (7353)
Jewish	0.1 (82)
Buddhist	0.1 (38)
Hindu	0.1 (32)
Other	1.7 (1153)
No affiliation	23.2 (15,147)
Don’t know/missing	1.1 (691)
Religious diversity index mean (min., max.)	0.42 (0.03, 0.76)
Self-rated health % (*n*)	
Very good	20.0 (13,065)
Good	39.5 (25,809)
Fair	29.3 (19,149)
Poor	9.0 (5857)
Very poor	1.9 (1224)
Not known	0.3 (199)

### Religiosity and Health: Individual Level

Overall, 60% (*n* = 38,874) of people had good or very good self-reported health; men were more likely than women to report good or very good health compared to fair, poor, or very poor (OR = 1.35, 95% CI 1.31–1.39, *p* < 0.0001) as were younger people (< 50 years) compared to older (≥ 50 years) (OR = 3.52, 95% CI 3.41–3.64, *p* < 0.0001) (Table 2).

**Table 2:** Socio-demographic and religiosity characteristics by self-reported health

	Self-rated health*		
	Very good or good	Fair	Poor or very poor
	(*n* = 38,874)	(*n* = 19,149)	(*n* = 7081)
Sex % (*n*)			
Female	56.7 (20,509)	30.9 (11,172)	12.4 (4514)
Male	63.6 (18,362)	27.6 (7977)	8.9 (2567)
Age % (*n*)			
< 50 years	72.4 (26,262)	23.0 (8348)	4.7 (1686)
≥ 50 years	43.8 (12,541)	37.5 (10,801)	18.7 (5395)
Education level % (*n*)			
Lower (elementary or incomplete secondary)	48.7 (9236)	33.2 (6290)	18.1 (3439)
Middle (complete secondary)	62.0 (18,360)	28.9 (8573)	9.1 (2697)
Upper (tertiary)	69.0 (10,699)	25.6 (3968)	5.4 (829)
Religious dimension, scale [Median, (IQR)]			
Attendance, 0–6	3 (0, 4)	3 (0, 4)	3 (0, 4)
Private prayer, 0–6	2 (0, 5)	4 (1, 6)	5 (1, 6)
Importance of religion, 0–15	10 (5, 13)	11 (6, 14)	12 (8, 14)
Belief in God, 0–4	3 (2, 4)	3 (2, 4)	3 (2, 4)

*IQR* Interquartile range

*Missing for 199 people

The country-specific odds ratio (OR) of more favourable health for a unit increase in the religion measure, from ordinal regression models adjusted for age, sex and education, are shown in Table 3 and graphically in Supplementary Figs. 1 to 4. ORs significantly greater than one reflect countries where individuals with greater religiosity are more likely to report more favourable health; ORs significantly less than one show countries where individuals with greater religiosity are less likely to report more favourable health.

**Table 3:** Odds ratio and 95% confidence interval (CI) of reporting more favourable health for a one unit increase in dimension with significant (*p* < 0.05) associations indicated as positive (bold) and negative (italics), by country (adjusted for age, sex, and education)

	Attendance*			Private prayer*			Importance of religion*			Belief in God*		
	OR	LCL	UCL	OR	LCL	UCL	OR	LCL	UCL	OR	LCL	UCL
Albania	*0.93*	*0.88*	*0.99*	1.00	0.96	1.06	1.00	0.98	1.03	1.04	0.95	1.13
Armenia	1.07	1.00	1.14	0.95	0.90	1.01	0.99	0.96	1.03	0.99	0.89	1.11
Austria	**1.09**	**1.02**	**1.17**	1.04	0.98	1.11	**1.04**	**1.01**	**1.07**	1.09	0.98	1.21
Azerbaijan	**1.11**	**1.03**	**1.19**	*0.78*	*0.71*	*0.85*	**1.04**	**1.00**	**1.07**	1.01	0.92	1.11
Belarus	0.96	0.90	1.03	*0.93*	*0.89*	*0.98*	*0.96*	*0.93*	*0.98*	0.92	0.84	1.01
Belgium	1.04	0.98	1.12	*0.94*	*0.89*	*0.99*	0.99	0.96	1.01	0.94	0.85	1.03
Bosnia-Herzegovina	**1.15**	**1.08**	**1.22**	**1.12**	**1.07**	**1.18**	**1.06**	**1.03**	**1.09**	1.08	0.97	1.20
Bulgaria	1.03	0.95	1.10	0.97	0.92	1.02	1.00	0.98	1.03	1.02	0.93	1.11
Croatia	**1.09**	**1.03**	**1.15**	0.99	0.95	1.05	1.02	0.99	1.05	1.10	1.00	1.22
Cyprus	**1.12**	**1.00**	**1.26**	0.94	0.86	1.03	0.94	0.87	1.00	1.07	0.83	1.37
Czech Republic	0.97	0.92	1.03	*0.91*	*0.87*	*0.96*	*0.97*	*0.95*	*0.99*	*0.92*	*0.85*	*0.99*
Denmark	1.05	0.96	1.14	0.95	0.89	1.01	*0.96*	*0.93*	*0.99*	0.99	0.90	1.08
Estonia	0.99	0.92	1.06	*0.94*	*0.90*	*0.99*	0.98	0.96	1.00	0.94	0.87	1.02
Finland	**1.11**	**1.02**	**1.22**	1.01	0.95	1.07	1.00	0.98	1.03	0.98	0.90	1.07
France	0.96	0.90	1.03	*0.93*	*0.88*	*0.98*	0.98	0.96	1.01	0.97	0.90	1.06
Georgia	**1.10**	**1.03**	**1.18**	1.00	0.95	1.05	1.02	0.98	1.07	0.95	0.78	1.16
Germany	**1.17**	**1.11**	**1.24**	**1.06**	**1.01**	**1.11**	**1.04**	**1.02**	**1.06**	**1.12**	**1.06**	**1.20**
Great Britain	**1.10**	**1.03**	**1.17**	0.98	0.93	1.03	1.01	0.99	1.04	**1.10**	**1.01**	**1.21**
Greece	**1.13**	**1.02**	**1.25**	1.05	0.98	1.12	1.01	0.97	1.05	1.18	1.00	1.39
Hungary	0.99	0.92	1.05	0.95	0.90	1.00	0.98	0.96	1.00	0.98	0.90	1.05
Iceland	**1.12**	**1.00**	**1.26**	1.07	0.99	1.16	**1.05**	**1.01**	**1.10**	**1.15**	**1.01**	**1.31**
Ireland	**1.13**	**1.02**	**1.25**	1.05	0.95	1.16	1.00	0.94	1.05	1.04	0.88	1.24
Italy	**1.07**	**1.00**	**1.14**	1.00	0.94	1.05	1.02	0.99	1.05	0.98	0.89	1.09
Kosovo	0.96	0.89	1.04	1.02	0.95	1.09	1.01	0.97	1.06	1.02	0.90	1.17
Latvia	0.99	0.92	1.06	*0.94*	*0.90*	*0.99*	*0.97*	*0.95*	*1.00*	0.95	0.85	1.05
Lithuania	1.01	0.94	1.10	0.96	0.90	1.02	0.98	0.95	1.01	1.01	0.92	1.11
Luxembourg	0.98	0.91	1.05	*0.94*	*0.89*	*1.00*	0.98	0.95	1.01	0.99	0.90	1.09
Macedonia	0.99	0.92	1.07	0.99	0.94	1.05	1.02	0.99	1.05	**1.24**	**1.11**	**1.38**
Malta	**1.12**	**1.05**	**1.20**	1.05	0.96	1.14	1.01	0.96	1.06	0.87	0.74	1.01
Moldova	1.06	0.98	1.15	*0.89*	*0.84*	*0.95*	0.97	0.94	1.01	0.91	0.82	1.02
Montenegro	1.02	0.95	1.09	0.99	0.94	1.04	1.02	1.00	1.05	1.01	0.92	1.11
Netherlands	1.06	1.00	1.12	0.98	0.93	1.02	1.00	0.98	1.03	1.02	0.94	1.12
Northern Cyprus	0.93	0.84	1.03	1.05	0.93	1.18	1.00	0.94	1.06	1.11	0.88	1.39
Northern Ireland	1.09	0.99	1.20	1.03	0.93	1.13	0.99	0.94	1.05	1.16	0.96	1.40
Norway	1.02	0.93	1.12	0.95	0.88	1.02	0.97	0.94	1.01	*0.88*	*0.79*	*0.99*
Poland	**1.11**	**1.03**	**1.20**	0.97	0.90	1.04	0.97	0.93	1.01	1.01	0.89	1.15
Portugal	1.01	0.95	1.07	0.95	0.90	1.00	*0.96*	*0.93*	*0.99*	0.90	0.81	1.00
Romania	1.04	0.96	1.12	0.96	0.89	1.02	1.00	0.96	1.04	1.02	0.91	1.15
Russian Federation	1.00	0.94	1.07	*0.92*	*0.88*	*0.97*	*0.97*	*0.95*	*1.00*	0.96	0.89	1.04
Serbia	1.06	0.99	1.13	1.01	0.96	1.06	1.01	0.98	1.04	1.11	1.00	1.23
Slovak Republic	0.98	0.93	1.03	*0.92*	*0.87*	*0.96*	*0.97*	*0.95*	*0.99*	0.95	0.87	1.02
Slovenia	1.05	0.98	1.11	0.98	0.93	1.03	0.98	0.95	1.01	0.99	0.90	1.09
Spain	1.00	0.94	1.07	0.96	0.91	1.02	1.00	0.96	1.03	1.05	0.95	1.17
Sweden	1.04	0.94	1.16	0.96	0.89	1.03	0.99	0.96	1.02	0.96	0.85	1.08
Switzerland	0.97	0.90	1.05	*0.93*	*0.87*	*0.99*	1.00	0.97	1.03	0.95	0.84	1.07
Turkey	**1.05**	**1.00**	**1.10**	**1.11**	**1.03**	**1.19**	**1.04**	**1.00**	**1.07**	1.13	0.95	1.33
Ukraine	**1.07**	**1.00**	**1.14**	0.99	0.95	1.04	1.00	0.98	1.03	1.01	0.92	1.10

*Analyses based on non-missing values as follows: attendance (*n* = 63,831), private prayer (*n* = 62,147), importance of religion (*n* = 64,524), belief in God (*n* = 63,918)

There was substantial variation in associations between dimensions of religiosity and health across the 47 countries, though a few countries showed remarkable consistency across all religiosity measures. For example, in Germany, increases in all four religiosity measures were associated with a greater likelihood of reporting more favourable health, whilst in Bosnia-Herzegovina, Iceland, and Turkey this was true for three out of the four measures (Table 3). Significant negative associations between self-reported health and three of the four religiosity measures are apparent in the Czech Republic (Table 3). No significant associations with health for any of the four dimensions were found in 14 countries (Armenia, Bulgaria, Hungary, Kosovo, Lithuania, Montenegro, Netherlands, Northern Cyprus, Northern Ireland, Romania, Serbia, Slovenia, Spain, Sweden), and no association with health for three of the four measures was found in an additional 20 countries.

For most countries, the association with self-reported health was greatest for frequency of attendance at religious services and lowest for belief in God. For 17 countries, increasing attendance was significantly associated with reporting more favourable health, although in one country (Albania) those with higher attendance were significantly less likely to report more favourable health (Table 3 and Supplementary Fig. 1). In only three countries (Bosnia-Herzegovina, Turkey, Germany) was increased private prayer significantly associated with a greater likelihood of more favourable health, whilst in 12 countries individuals with higher private prayer frequency were significantly less likely to report more favourable health (Table 3 and Supplementary Fig. 2). In six countries, a greater importance of religion was significantly associated with more favourable health, whilst in seven countries individuals attaching a greater importance to religion were less likely to report more favourable health (Table 3 and Supplementary Fig. 3). Belief in God was significantly associated with reporting more favourable health in five countries and reporting less favourable health in two countries (Czech Republic, and Norway) (Table 3 and Supplementary Fig. 4).

### Religiosity and Healthy Life Expectancy

Healthy life expectancy at age 20 (HLE20) for the 45 countries with life tables ranged from 14.9 years (Russian Federation) to 50.3 years (Ireland) in men, and 13.5 years (Russian Federation) to 48.8 years (Ireland) in women. In general, countries with higher life expectancy had higher HLE20, this relationship being stronger in males (*r* = 0.93) than females (*r* = 0.84) (Supplementary Fig. 5). However, despite the strong relationship between HLE20 and life expectancy, the proportion of remaining life spent healthy at age 20 varied considerably between countries, from 35.3% (Russian Federation) to 86.6% (Ireland) for men, and from 24.9% (Russian Federation) to 78.4% (Denmark) for women, with a median of 62.9% for men and 55.2% for women.

The associations between individual measures of religiosity, summarised for each country by the mean, and LE20 and HLE20 are shown in Table 4. The coefficients for all four measures of religiosity with LE20 and HLE20 in all models were negative (though not always significantly different from zero) suggesting inverse relationships between each measure and LE20 and HLE20. While attendance had the strongest association with good health at the individual level, at the country level no associations were evident between attendance at religious services and LE20 or HLE20 in any model. Private prayer and importance of religion were significantly inversely associated with both LE20 and HLE20, after adjustment for education and Gini coefficient, but only in women; these associations remained significant after further adjustment for religious diversity. Belief in God was also significantly inversely associated with LE20 and HLE20 after adjustment for education, Gini coefficient and religious diversity. For men, no associations were evident between LE20 or HLE20 and any of the dimensions of religiosity.

**Table 4:** Coefficients from regression models of each religious dimension (summarised by the mean) as a predictor of life expectancy at age 20 (LE20) and healthy life expectancy at age 20 (HLE20), unadjusted and adjusted, for 41 countries (significant associations in bold)

Dimension	Sex	Model 1*	*p* value	Model 2*	*p* value	Model 3*	*p* value
		β (95% CI)		β (95% CI)		β (95% CI)	
LE20**							
Attendance	M	− 0.67 (− 2.58, 1.24)	0.48	− 0.50 (− 2.47, 1.48)	0.61	− 1.38 (− 4.19, 1.44)	0.33
	F	− 0.97 (− 2.25, 0.31)	0.13	− 0.88 (− 2.13, 0.38)	0.16	− 1.07 (− 2.66, 0.53)	0.18
Prayer	M	− 0.66 (− 2.02, 0.71)	0.33	− 0.48 (− 1.86, 0.90)	0.49	− 0.94 (− 2.69, 0.81)	0.28
	**F**	− **0.94 (**− **1.84,** − **0.05)**	**0.04**	− **1.13 (**− **2.03,** − **0.24)**	**0.01**	− **1.56 (**− **2.68,** − **0.44)**	**0.01**
Importance of religion	M	− 0.45 (− 1.08, 0.18)	0.15	− 0.30 (− 0.99, 0.38)	0.37	− 0.64 (− 1.54, 0.27)	0.16
	F	− **0.58 (**− **1.01,** − **0.15)**	**0.01**	− **0.67 (**− **1.12,** − **0.22)**	< **0.01**	− **0.94 (**− **1.49,** − **0.39)**	< **0.01**
Belief in God	M	− 1.32 (− 3.77, 1.14)	0.29	− 0.97 (− 3.57, 1.62)	0.45	− 2.03 (− 5.41, 1.36)	0.23
	F	− 1.62 (− 3.53, 0.28)	0.09	− 1.93 (− 3.95, 0.09)	0.06	− **2.64 (**− **5.27,** − **0.02)**	**0.05**
HLE20**							
Attendance	M	− 0.32 (− 4.04, 3.41)	0.86	− 0.28 (− 4.20, 3.64)	0.88	− 1.80 (− 7.41, 3.81)	0.52
	F	− 3.12 (− 7.15, 0.90)	0.12	− 2.88 (− 7.00, 1.25)	0.17	− 4.93 (− 10.09, 0.22)	0.06
Prayer	M	− 1.03 (− 3.77, 1.71)	0.45	− 0.95 (− 3.79, 1.90)	0.50	− 2.13 (− 5.72, 1.47)	0.24
	F	− **2.85 (**− **5.73, 0.04)**	**0.05**	− **3.37 (**− **6.43,** − **0.32)**	**0.03**	− **5.52 (**− **9.20,** − **1.84)**	< **0.01**
Importance of religion	M	− 0.61 (− 1.87, 0.64)	0.33	− 0.48 (− 1.85, 0.89)	0.48	− 1.20 (− 3.02, 0.62)	0.19
	**F**	− **1.72 (**− **3.09,** − **0.35)**	**0.02**	− **1.93 (**− **3.45,** − **0.41)**	**0.01**	− **3.24 (**− **5.06,** − **1.42)**	< **0.01**
Belief in God	M	− 2.12 (− 6.99, 2.76)	0.38	− 2.20 (− 7.43, 3.02)	0.40	− 4.97 (− 11.71, 1.76)	0.14
	F	− 5.61 (− 11.62, 0.39)	0.07	− 6.57 (− 13.22, 0.08)	0.05	− **11.41 (**− **19.66,** − **3.15)**	**0.01**

*Model 1: unadjusted, Model 2: adjusted for education level and Gini coefficient, Model 3: adjusted for education level, Gini coefficient and index of religious diversity

**Standard regression models for LE20; meta-regression models for HLE20

### Sensitivity Analyses

The response rates for the EVS2008 varied considerably by country. Eight countries had a response rate of below 50%: Luxembourg (32.0%), Russian Federation (35.9%), Greece (36.8%), France (40.0%), Switzerland (44.0%), Sweden (45.7%), Moldova (47.1%), and Ireland (47.9%); the maximum response rate was from Azerbaijan (93.5%). Adjusting the meta-regression by the response rate of each country slightly attenuated the coefficients, but left the results unchanged (Supplementary Table 2).

## Discussion

In this cross-sectional study, we explored two dimensions of religiosity that have been examined commonly alongside health—frequency of attendance and frequency of private prayer—and a further two dimensions less well researched—importance of religion and belief in God. We found that increased attendance had the strongest positive relationship with self-rated health, individuals with higher frequency of attendance being more likely to report good health in 17 countries. However, individuals with higher frequency of private prayer were significantly less likely to report good health in 12 countries. In country-level analyses higher mean religiosity, as measured by private prayer or importance of religion, was significantly associated with lower LE20 and HLE20, after adjustment for education, inequality and religious diversity, but only in women. In fully adjusted models, we also found evidence of a relationship between higher mean levels of belief in God and lower HLE20, but again only in women. There was no evidence of significant associations between any of the four religiosity dimensions and either LE20 or HLE20 in men, in unadjusted, or fully adjusted models. Additionally, these negative relationships between health and both private prayer and importance of religion were evident within countries, with more countries showing a negative relationship than a positive one.

Previous research has focussed on the older population. Our recent literature review of the relationship between religiosity/spirituality and health in later life also demonstrates the considerable evidence linking religiosity to lower mortality and to better health, including physical and mental health, as well as more specific health outcomes such as cardiovascular disease, stroke, cancer, pain, and length of hospitalisation (Zimmer et al., 2016). Although there has been research investigating the association between religiosity and health across a wide range of countries (Zimmer et al., 2018), including Europe (Ahrenfeldt et al., 2017; Nicholson et al., 2009, 2010), our study is the first to consider mortality and health simultaneously in healthy life expectancy across multiple countries. Previous studies on this topic were carried out only in single countries, Taiwan (Hidajat et al., 2013; Zimmer et al., 2020) and Sweden (Schön et al., 2011). At an individual level, our finding of a stronger positive relationship between health and religiosity for attendance than any other dimension confirmed results of a study that found significant positive associations between attendance and health in Finland, Greece and Ireland, and a negative relationship between prayer and health in Estonia (Nicholson et al., 2010). In our study, a negative relationship between healthy life expectancy and private prayer was evident only in women, which again mirrors other European analyses albeit in prevalence of self-rated health rather than healthy life expectancy (Nicholson et al., 2010). Associations between attendance and self-rated health have been found to be stronger in men (Nicholson et al., 2009), though we found no evidence with healthy life expectancy in men or women. Australian data have also shown high levels of faith and attendance to be associated with worse health (Bernardelli et al., 2020).

At an individual level, three mechanisms are commonly considered as being important ones through which religion may positively affect health: quantity and quality of network support; more salutary behaviours, such as avoidance of smoking or alcohol; and improving psychological well-being through means like stress reduction and coping mechanisms (Kevin & Peter, 2001). The social, cultural, political, and economic systems of a country may also have an effect on an individual’s health and mortality and therefore with the healthy life expectancy of a country. To partly account for this, we included in our analyses an index of religious diversity based on the distribution of religions within each country (Zimmer et al., 2018).

In some studies, Gross Domestic Product (GDP) has been included as a covariate in models to control for economic development. However, there is evidence that over the past century, religious change, specifically the rise in secularisation, has preceded economic growth development (Ruck et al., 2018). When GDP was included in our models (data not shown), model coefficients for all religiosity measures were attenuated, and the relationships between prayer and importance of religion, and HLE20 in women were no longer significant. This gives credence to GDP being a mediator between religiosity and healthy life expectancy, particularly as there is a strong relationship between higher GDP and higher HLE (Jagger et al., 2008). It is, nevertheless, impossible from the present analysis to establish cause.

### Strengths and Limitations

Strengths of our study include the following: the high quality, nationally representative data; the large number and variety of countries; our control of education level, economic inequality and religious diversity; as well as using the four dimensions of religiosity: attendance, private prayer, importance of religion, and belief in God, especially since previous studies have found that the relationship between religiosity and well-being depends on the measure of religiosity used as well as the national context (Lun & Bond, 2013). Our study does have limitations. Despite the EVS being longitudinal, we had to calculate HLE using national life tables and prevalence of good self-rated health as the EVS does not have linked mortality data. Given the cross-sectional nature of our analyses, we could only explore associations and not causal relationships. The largely Christian sample limited our ability to make inferences about other religions, and as the sample sizes were relatively small, we could not explore differences by religious group within countries. Neither did we adjust for religious denomination in the individual-level analyses of the relationship between self-rated health and religiosity as (a) at an individual-level denomination did not capture religious diversity, and (b) denomination is likely to be highly correlated with most of the religiosity measures. In addition, the response rates varied considerably between the countries, although a sensitivity analysis provided no evidence that this affected our conclusions.

In conclusion, this is the first study, albeit cross-sectional, exploring the relationship between different dimensions of religiosity and healthy life expectancy in a large number of European countries. We found evidence that greater levels of private prayer, importance of religion, and belief in God, at a country level, were associated with lower healthy life expectancy at age 20, after adjustment for education, inequality, and religious diversity, but only in women. These findings may contribute to the inequalities in healthy life years found across Europe, but should be confirmed in longitudinal analyses.

## Supplementary Information

Below is the link to the electronic supplementary material.

Supplementary file1 (DOCX 484 KB)

## Data Availability

The European Values Survey data and life tables are publicly available.
