# Supplementary material for: Are Religiosity and Spirituality Related to Self-Reported Health Expectancy? An Analysis of the European Values Survey
Source: J Relig Health. 2021 Jul 20;61(3):2590–604. doi: 10.1007/s10943-021-01348-w (PMC9142421; doi:10.1007/s10943-021-01348-w)
Supplement: Supplementary file 1 — Supplementary file1 (DOCX 484 KB) [file 10943_2021_1348_MOESM1_ESM.docx]

**Supplementary material for**

**Are religiosity and spirituality related to self-reported health expectancy? An analysis of the European Values Survey**

**Supplementary Table 1. Individual questions, responses and scoring underlying the four dimensions of religiosity: frequency of attendance at religious services, frequency of private prayer, importance of religion, and belief in God (European Values Survey 2008)**

|  | Question | (Score) Response |  |
| --- | --- | --- | --- |
| Dimension |  |  | Total Score (range) |
| Attendance | Apart from weddings, funerals and christenings, about how often do you attend religious services these days? | 1. never, practically never 2. less often 3. once a year 4. only on specific holy day 5. once a month 6. once a week 7. more than once week |  |
|  |  |  | *0-6* |
| Prayer | How often do you pray to God outside of religious services? | 1. never 2. less often 3. several times a year 4. at least once a month 5. once a week 6. more than once a week 7. every day |  |
|  |  |  | *0-6* |
| Importance of religion | How important is religion in your life? | 1. not at all important 2. not important 3. quite important 4. very important |  |
|  | Do you personally think it is important to hold a religious service for any of the following events?  Birth  Marriage  Death | (1) Yes (0) No  (1) Yes (0) No  (1) Yes (0) No |  |
|  | And how important is God in your life? | 1. not at all important –   (9) very important |  |
|  |  |  | *0-15* |
| Belief in God | Do you believe in God? | (1) Yes (0) No |  |
|  | Which of these statements comes closest to your beliefs? | (3) there is a personal God.  (2) there is some sort of spirit or life force  (1) I don’t really know what to think  (0) I don’t really think there is any sort of spirit, God or life force |  |
|  |  |  | *0-4* |

**Supplementary Table 2. Coefficients from meta-regression models of each religious dimension (summarised by the mean) as a predictor of healthy life expectancy at age 20 (HLE20), with and without adjustment for response rate (significant associations in bold)**

|  |  | Model 3* |  | Model 3A* |  | Model 3B* |  |
| --- | --- | --- | --- | --- | --- | --- | --- |
| Dimension | Sex | β (95% CI) | *p*-value | β (95% CI) | *p*-value | β (95% CI) | *p*-value |
| *HLE20* |  |  |  |  |  |  |  |
| Attendance | M | -1.80 (-7.41, 3.81) | 0.52 | -1.70 ( -7.37, 3.97) | 0.55 | -0.09 (-5.60, 5.41) | 0.97 |
|  | F | -4.93 (-10.09, 0.22) | 0.06 | -4.85 ( -10.07, 0.37) | 0.07 | -3.92 (-9.17, 1.32) | 0.14 |
| Prayer | M | -2.13 (-5.72, 1.47) | 0.24 | -2.04 ( -5.68, 1.60) | 0.26 | -1.16 (-4.69, 2.37) | 0.51 |
|  | **F** | **-5.52 (-9.20, -1.84)** | **<0.001** | **-5.44 ( -9.20, -1.69)** | **0.01** | **-4.75 (-8.60, -0.90)** | **0.02** |
| Importance of religion | M | -1.20 (-3.02, 0.62) | 0.19 | -1.11 ( -2.98, 0.76) | 0.24 | -0.65 (-2.47, 1.17) | 0.47 |
|  | **F** | **-3.24 (-5.06, -1.42)** | **<0.001** | **-3.24 ( -5.13, -1.35)** | **<0.001** | **-2.91 (-4.85, -0.97)** | **<0.001** |
| Belief in God | M | -4.97 (-11.71, 1.76) | 0.14 | -4.65 ( -11.65, 2.36) | 0.19 | -2.21 (-9.29, 4.86) | 0.53 |
|  | **F** | **-11.41 (-19.66, -3.15)** | **0.01** | **-11.43 ( -20.09, 2.76)** | **0.01** | **-9.57 (-18.74, -0.41)** | **0.04** |

*Model 3: as Table 4, adjusted for education, Gini index, index of religious diversity and including Germany; Model 3A: as Model 3 but excluding Germany; Model 3B: as Model 3A further adjusted for response rate

**Supplementary Figure 1. Odds ratio and 95% confidence interval (CI) of reporting more favourable health for a one unit increase in attendance, by country (adjusted for age, sex and education)**

**Supplementary Figure 2. Odds ratio and 95% confidence interval (CI) of reporting more favourable health for a one unit increase in frequency of private prayer, by country (adjusted for age, sex and education)**

**Supplementary Figure 3. Odds ratio and 95% confidence interval (CI) of reporting more favourable health for a one unit increase in importance of religion, by country (adjusted for age, sex and education)**

**Supplementary Figure 4. Odds ratio and 95% confidence interval (CI) of reporting more favourable health for a one unit increase in belief in God, by country (adjusted for age, sex and education)**

**Supplementary Figure 5: Healthy Life Expectancy and Total Life Expectancy at age 20 by country and sex**

**Men**


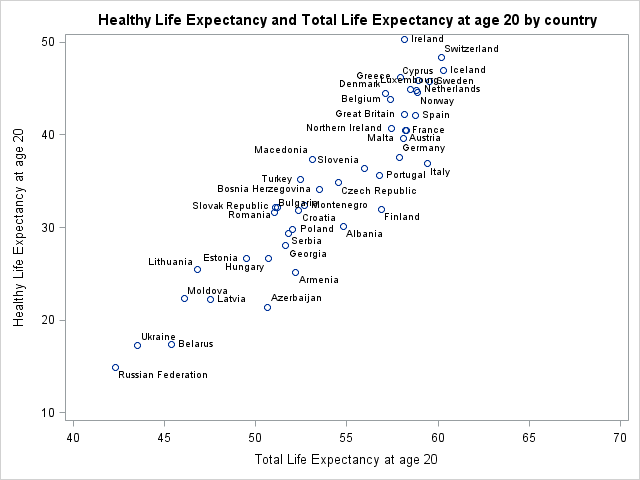

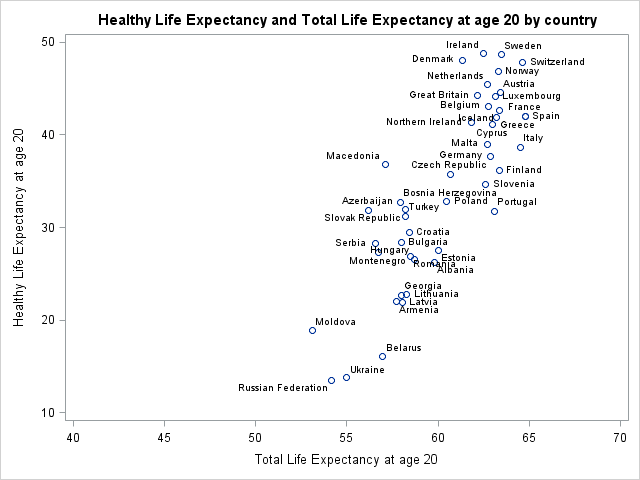


**Women**
